# Topologic and Hemodynamic Characteristics of the Human Coronary Arterial Circulation

**DOI:** 10.3389/fphys.2019.01611

**Published:** 2020-01-23

**Authors:** Janina C. V. Schwarz, Monique G. J. T. B. van Lier, Jeroen P. H. M. van den Wijngaard, Maria Siebes, Ed VanBavel

**Affiliations:** Department of Biomedical Engineering and Physics, Amsterdam UMC, University of Amsterdam, Amsterdam, Netherlands

**Keywords:** human, coronary circulation, hemodynamics, myocardial perfusion, modeling, branching patterns, scaling laws

## Abstract

**Background:**

Many processes contributing to the functional and structural regulation of the coronary circulation have been identified. A proper understanding of the complex interplay of these processes requires a quantitative systems approach that includes the complexity of the coronary network. The purpose of this study was to provide a detailed quantification of the branching characteristics and local hemodynamics of the human coronary circulation.

**Methods:**

The coronary arteries of a human heart were filled post-mortem with fluorescent replica material. The frozen heart was alternately cut and block-face imaged using a high-resolution imaging cryomicrotome. From the resulting 3D reconstruction of the left coronary circulation, topological (node and loop characteristics), topographic (diameters and length of segments), and geometric (position) properties were analyzed, along with predictions of local hemodynamics (pressure and flow).

**Results:**

The reconstructed left coronary tree consisted of 202,184 segments with diameters ranging from 30 μm to 4 mm. Most segments were between 100 μm and 1 mm long. The median segment length was similar for diameters ranging between 75 and 200 μm. 91% of the nodes were bifurcations. These bifurcations were more symmetric and less variable in smaller vessels. Most of the pressure drop occurred in vessels between 200 μm and 1 mm in diameter. Downstream conductance variability affected neither local pressure nor median local flow and added limited extra variation of local flow. The left coronary circulation perfused 358 cm^3^ of myocardium. Median perfused volume at a truncation level of 100 to 200 μm was 20 mm^3^ with a median perfusion of 5.6 ml/min/g and a high local heterogeneity.

**Conclusion:**

This study provides the branching characteristics and hemodynamic analysis of the left coronary arterial circulation of a human heart. The resulting model can be deployed for further hemodynamic studies at the whole organ and local level.

## Introduction

The coronary arterial circulation consists of a myriad of vessel segments, starting at the main stem and right coronary artery and repeatedly branching toward the smallest arterioles that connect to the capillary bed. This system, covering around a 500-fold range in diameters in humans, normally allows for adequate matching of local perfusion to the oxygen needs. Thus, coronary autoregulation ensures by adjusting vasomotor tone that local perfusion at rest increases with, among others, oxygen demand, and is relatively insensitive to changes in systemic pressure (Mosher et al., 1964; Goodwill et al., 2017). It is generally believed that also the coronary artery structure adapts to allow for optimal perfusion capacity, although the concepts and mechanisms here are far less clear (Seiler et al., 1992; Kassab, 2005). This system is challenged in coronary artery disease (CAD), where proximal stenoses cause flow impairment. In addition, downstream coronary arteries and arterioles may also be affected by CAD, resulting in endothelial dysfunction and, consequently, impaired regulatory capacity and reduced ability for structural adaptation.

Many processes have been identified that contribute to functional and structural regulation in the coronary circulation. For acute autoregulation, these include effects of local metabolites released from the cardiomyocytes, the myogenic response to changes in local pressure, and flow-dependent dilation (Dole, 1987). In addition, conducted vasomotor responses may further integrate the local responses (Palao et al., 2018). Structural adaption may include hypoxia-driven angiogenesis, flow-dependent remodeling, and pressure-induced changes in wall-to-lumen ratio (Hoefer et al., 2013; Silvestre et al., 2013; Zimarino et al., 2014). We also demonstrated in a range of *in vitro* and *in vivo* settings that vasomotor tone itself is a drive for structural changes in arterial caliber (Bakker et al., 2002; VanBavel et al., 2006).

It has long been realized that proper understanding of the interplay of all of these processes requires a quantitative systems approach that includes the complexity of the coronary network. Accordingly, several previous studies provided detailed descriptions of such networks, based mostly on porcine coronary arterial casts (VanBavel and Spaan, 1992; Kassab et al., 1993; Kassab and Fung, 1994; Kaimovitz et al., 2010). Yet, data on human coronary branching patterns are extremely scarce (Zamir and Chee, 1986; Zamir, 1999; van Lier et al., 2016).

The purpose of the current study therefore was to provide a detailed quantification of the branching characteristics of the human coronary circulation. These data were obtained from a human heart in which coronary arteries were filled with casting material, followed by sectioning in a 3D imaging cryomicrotome, 3D reconstruction, and *post hoc* image processing (Spaan et al., 2005). Our analysis included topological (e.g., node and loop characteristics), topographic (diameters and length of segments) and geometric (position) data along with predictions of local hemodynamics (pressure and flow). These data allow evaluation of the relevance of previous animal studies and provide a base for a systems analysis of human coronary flow regulation.

## Materials and Methods

### Human Heart

The data in this study were derived from a post-mortem human heart obtained at the Department of Pathology of the Academic Medical Center, University of Amsterdam, Netherlands. Heart weight was 330 gram. The patient was an 84-year-old female suffering from amyotrophic lateral sclerosis (ALS). Cause of death was listed as euthanasia. The patient history included atrial tachycardia, mitral stenosis, abdominal aortic aneurysm, atherosclerosis, and hypertension. However, the patient had never suffered any major cardiovascular events, and the heart had a normal appearance, without evidence of contracture. The patient’s relatives gave written consent to use this heart for research.

### Vascular Cast and Imaging

**FIGURE 1:**
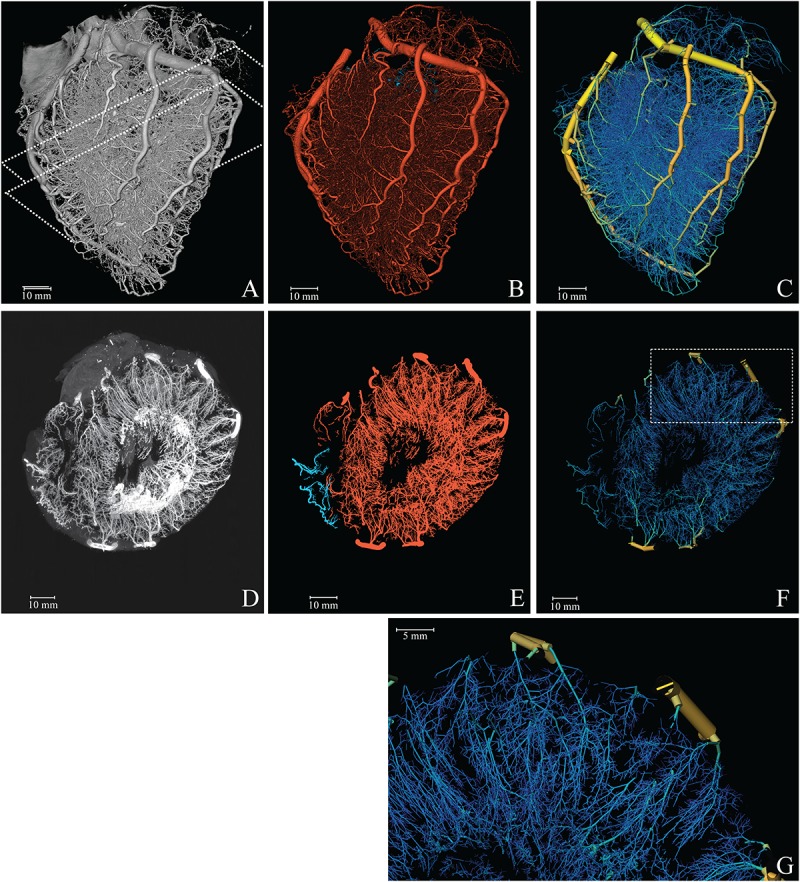
Reconstruction of the coronary vasculature. **(A)** Raw data. Dashed lines indicate the 1 cm slab (transversal, parallel to the cutting plane) used for the MIP. **(B)** Segmented vasculature. **(C)** Reconstructed tree with color coded diameter (yellow = 4 mm, blue = 50 μm). **(D)** 1 cm MIP of raw data. **(E)** 1 cm MIP of segmented data (red, left; blue, right circulation). **(F)** 3D visualization of a 1 cm slab parallel to the cutting plane. **(G)** Magnification of the reconstructed vasculature (area indicated in **F**).

A 3D-representation of the coronary vasculature was obtained utilizing the cryomicrotome imaging procedure described previously (Spaan et al., 2005; van Horssen et al., 2014). In brief, after removing the heart, the left circumflex, left anterior descending artery, and right coronary artery were cannulated, flushed with calcium-free buffer and thereafter filled with fluorescent vascular cast material (UV-Blue, VasQtec, Switzerland, suspended in Batson’s no. 17, Polysciences, United States; infusion pressure 90 mmHg). The vascular filling protocol was optimized to fill down to arterioles of around 15 μm in diameter to avoid background fluorescence via capillary filling. After the cast material had hardened, the heart was suspended in a carboxymethylcellulose sodium solvent (Brunschwig Chemie, Netherlands) blackened with 5% Indian ink (Royal Talens, Netherlands) and frozen at −20°C. To acquire a 3D digital reproduction, the frozen sample was sectioned with a slice thickness of 30 μm, matching the in-plane image resolution. After every slice, the remaining block surface, rather than the slice itself, was imaged twice for two optical settings. This automated procedure thereby yielded two co-registered image stacks, each containing 4200 4096 × 4096 16-bit images: a stack with reflection images and a stack of fluorescent images optimized for the vascular cast (excitation, 365 nm and emission, 505 nm) (Figure 1A).

### Vessel Segmentation and Quantification

As a next step, the topological tree was extracted from the digital reproduction of the fluorescent vascular cast. For this, dark current artifacts were eliminated and optical blurring was corrected by deconvolving the images with a system-specific point-spread function (Matlab; The MathWorks Inc., Natick, MA, United States) (Schwarz et al., 2017). Subsequently, the vessels were segmented (Figure 1B) in three steps. First, the major coronary arteries were manually segmented in Amira (FEI Visualization Sciences Group, France) using a fourfold down-sampled image stack. Secondly, arterioles and arteries were enhanced by multi-scale vesselness filtering. For vessels smaller than 300 μm the original resolution was used; larger vessels were enhanced at half the original resolution. Thirdly, the centerline representation of the entire arterial vasculature was obtained by thresholding the result of the second step and subsequent merging with the skeleton of the manually segmented large vessels from step 1. The resulting image stack was up-sampled to full resolution, after which the centerlines were obtained by 3D-skeletonization (Lee et al., 1994). We visually inspected the results and compared these with the original image data. Obvious artifacts resulting from cast interruption or cutting debris were corrected.

Every point on a centerline was classified according to the number of neighboring points. Points with a single neighbor were classified as terminal node. Points with two neighbors were considered to be mid-segmental points. Points with three or more neighbors in the skeleton reflected internal nodes connecting multiple segments.

The diameter at each point was estimated using a full width at half maximum algorithm on the normal plane. Segments were defined as the centerline path between two nodes. Internal segments connect two internal nodes, while terminal segments connect an internal and terminal node. Segment diameters were determined as the mean of the diameters of the segment’s mid-segment points. Spurious terminal segments and triangular loops were eliminated based on quality measures for the diameter, including the condition that the segment length should be larger than the segment diameter. Diameters of internal segments that were underestimated, e.g., due to cast artifacts, were corrected by interpolation from diameters of neighboring segments. The results were stored in graph notation, a means to study relations and processes in networks, representing the vasculature as a set of nodes and cylindrical segments with associated information on its characteristics and its neighboring nodes and segments. For every node, the proximity to its coronary root was calculated. Segment description included their path length, mean diameter, as well as myocardial region (Figure 1C).

In order to study vessel branching, bifurcations were identified. For every bifurcation, the three connected segments were classified into mother and daughter segments based on flow direction.

The symmetry (S) of the daughters was defined as the ratio of the smaller (*d*_*S*_) to the larger (*d*_*L*_) diameter.

(1) $$S=d_{S}/d_{L}$$

As a second parameter, the relation between mother (M) and her daughters was represented by the increase in total cross-sectional area:

(2) $$A=\frac{d_{L}^{2}+d_{S}^{2}}{d_{M}^{2}}$$

For analysis, segments, and bifurcations were grouped into ten classes based on (mother) diameter (*d*_M_).

### Extension of the Measured Tree Toward the Terminal Microcirculation

The extracted coronary vascular network terminates at 30 μm segments, since this was the resolution of the imaging technique, or at larger diameters where cast material had insufficiently filled the arterial bed. In order to estimate hemodynamic properties of the human coronary circulation, extrapolation toward the terminal arterioles is required. Notably, distributions of downstream conductances distal to the extracted end segments are needed. These were obtained by simulating segments smaller than 30 μm based on interpolation and extrapolation of the extracted vascular network. These *in silico* trees were generated as follows. Starting with an initial segment with a diameter between 300 and 400 μm, two daughter segments were created with symmetry stochastically drawn from the symmetry distribution observed in this human heart coronary network for segments of the same diameter class. Their diameters were set to match the area growth for their symmetry and mother diameter as expected from the data. Segment lengths were randomly assigned based on the segment-length-to-diameter distribution of their diameter class. For diameters below 30 μm, extrapolated branching characteristics were used. This procedure was iterated until the capillary domain (5.0–7.5 μm) was reached. For every segment, the total conductance of its distal network was calculated. Fifty trees in total were generated for this purpose, resulting in a distribution of downstream conductances for segments in the range between 30 μm and 400 μm. These predictions were then imputed to the recorded tree. Finally, hemodynamics in the extracted coronary tree was determined for 1000 simulations of the stochastic terminal conductances.

### Computational Hemodynamic Modeling

Blood was modeled as an incompressible, isotropic homogeneous fluid. To account for the Fåhraeus-Lindqvist effect, an empirical diameter-dependent relationship derived by Pries et al. was used to model blood viscosity (μ), assuming a constant hematocrit *H*_*d*_ = 0.4 (Pries et al., 1994; Secomb and Pries, 2013):

(3) $$\mu =\left[1+\left(\mu_{0.45}^{{}^{*}}-1\right)\,\frac{{\left(1-H_{d}\right)}^{C}-1}{{\left(1-0.45\right)}^{C}-1}\,{\left(\frac{d}{d-1.1}\right)}^{2}\right]\times {\left(\frac{d}{d-1.1}\right)}^{2}$$

with

$$\mu_{0.45}^{{}^{*}}=6\,e^{-0.085\,d}+3.2-2.44\,{\text{e}}^{-0.06\,d^{0.645}}$$

$$C=\left(0.8+{\text{e}}^{-0.075\,\text{d}}\right)\,\left(-1+\frac{1}{1+10^{-11}\,d^{12}}\right)+\frac{1}{1+10^{-11}\,d^{12}}$$

The fully developed, laminar axisymmetric steady-state flow through a segment was modeled based on Poiseuille’s law including the above Fåhraeus-Lindqvist effect. For a segment with nodes i and j, the flow (Q_i→j_) through and the conductance (G_ij_) of the segment are:

(4) $$Q_{i\to j}=G_{i\,j}\,\left(P_{i}-P_{j}\right)$$

(5) $$G_{i\,j}=\frac{\pi \,d_{i\,j}^{4}}{128\,\mu_{i\,j}\,l_{i\,j}}$$

where P_i_ is the pressure at node i, μ_ij_ is the blood viscosity, d_ij_ is the diameter and l_ij_ the length of the segment between nodes i and j. Applying Kirchhoff’s current law, i.e., conservation of mass at every junction, ∑_i ∈ Nj_*Q*_i→j_ = 0, where N_j_ is the set of neighbor nodes, yields a system of linear equations that can be solved via matrix inversion for a given set of flow or pressure boundary conditions. We used a coronary inlet pressure of 90 mmHg and capillary pressure of 20 mmHg. Wall shear stress was estimated under the premise of non-accelerating flow such that the frictional forces with the wall balance the force from the pressure gradient in every segment (Munson et al., 2013).

(6) $$\tau_{i\,j}=\frac{\left(P_{i}-P_{j}\right)\,d_{i\,j}}{4\,l_{i\,j}}$$

Perfusion was derived by relating the flow through 100–200 μm segments to the weight of their perfusion territory. The perfusion territories were determined in 3D by Voronoi tessellation. Voxels within the heart belong to the Voronoi cell of the closest terminal node. The union of all Voronoi cells perfused by a single 100–200 μm segment was taken as its perfusion territory.

### Statistical Analysis

The difference between two daughter symmetry classes was tested using a Mann Whitney *U*-test. Differences between multiple diameter classes and myocardial regions were tested using a one-way Kruskal-Wallis analysis followed by a Dunn’s multiple comparison test. A two-way ANOVA followed by Bonferroni’s correction for multiple comparisons was employed to assess differences in area growth. Statistical analyses were performed using GraphPad Prism (GraphPad Software, Inc., La Jolla, CA, United States). Least-squares regression in Matlab was used for testing the agreement between hemodynamic results, for analyzing the relation between diameter, symmetry, and area growth (linear fit) and for determining the power laws (non-linear fit). A *p* < 0.05 was considered statistically significant.

## Results

### Coronary Arterial Topology

Figure 1A provides a 3D visualization of the coronary arterial cast (raw data), as acquired with the imaging cryomicrotome, while Figures 1B,C show the results of the segmentation in single color and diameter-encoded color, respectively. Figures 1D–G provide maximum intensity projections of a transversal section and a magnification. Visual inspection of the raw data revealed nearly complete filling of the arterial tree down to 30 μm, covering the expected perfusion territory. Vascular density appeared highest in the subendocardium and lowest in the subepicardium. While Figure 1 depicts both the left and right coronary circulation, the remainder of the study focused on the left coronary circulation.

The reconstructed left coronary tree consisted of 202,184 segments with diameters ranging from 30 μm in small arterioles to 4 mm for the epicardial arteries. Table 1 gives an overview of distribution of these segments over diameter classes and myocardial regions. Taking the class sizes into account, the data show increased density of smaller segments, as expected, which levels off below 60 μm diameter. We attribute this to incomplete filling of vessels smaller than 60 μm. Terminal segment density increased strongly from subepicardium to subendocardium, in accordance with visual inspection of the transversal sections of Figure 1. The distributions of terminal segment diameters in the three myocardial regions were very comparable, indicating that the increased subendocardial density is not a result of better filling of the distal vessels in this region.

**TABLE 1:** Segment distribution.

	**Number of segments (n, %)**		**LV density of terminal segments (n/cm^3^, %)**		
**Diameter class**	**All**	**Terminal**	**Subepicardium**	**Midmyocardium**	**Subendocardium**
30–45 μm	8,047 (4.0)	6,695 (6.6)	12.2 (7.2)	20.9 (5.8)	34.9 (7.3)
45–60 μm	22,204 (11.0)	20,760 (20.6)	37.4 (22.2)	65.5 (18.3)	104.2 (21.9)
60–75 μm	32,500 (16.1)	27,545 (27.3)	46.2 (27.4)	90.3 (25.2)	136.3 (28.6)
75–90 μm	33,605 (16.6)	22,872 (22.7)	38.0 (22.6)	83.0 (23.2)	106.8 (22.4)
90–105 μm	27,816 (13.8)	12,860 (12.7)	21.9 (13.0)	52.0 (14.5)	50.2 (10.5)
105–120 μm	19,444 (9.6)	5,428 (5.4)	7.9 (4.7)	23.9 (6.7)	21.6 (4.5)
120–150 μm	24,598 (12.2)	3,274 (3.2)	3.4 (2.0)	15.3 (4.3)	14.8 (3.1)
150–200 μm	19,718 (9.8)	1,204 (1.2)	1.1 (0.7)	5.5 (1.5)	6.2 (1.3)
200–400 μm	12,665 (6.3)	321 (0.3)	0.3 (0.2)	1.5 (0.4)	1.6 (0.3)
≥400 μm	1,587 (0.8)	2 (0.0)	0.0 (0.0)	0.0 (0.0)	0.0 (0.0)
Total	202,184 (100.0)	100,961 (100.0)	168.4 (100.0)	357.8 (100.0)	476.6 (100.0)
Diameter. median (μm)	92.3	72.4	71.2	75.4	70.6
Diameter. interdecile range (μm)	54.3–179	47.8–105	47.4–101	48.9–110	46.7–103
Length. median (μm)	441	443	473	453	391

The reconstructed coronary network was not a simple tree with only bifurcations. Rather, 91% of the nodes were bifurcations, 8% were trifurcations (connecting four segments) and less than 1% connected more than four segments. The network also contained arcades or loops: an analysis based on graph theory revealed 3,202 such loops.

**FIGURE 2:**
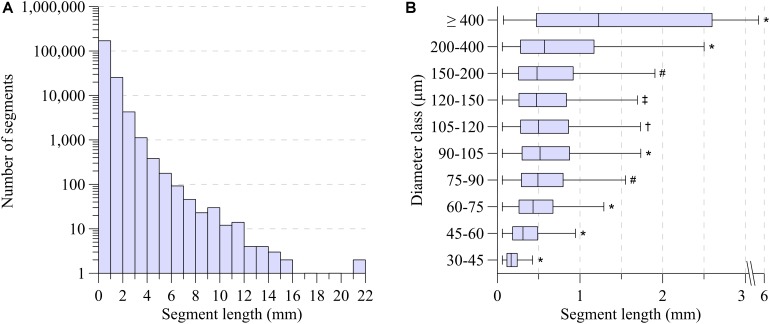
**(A)** Segment length distribution. **(B)** Segment length-diameter relation for ten diameter classes. Boxplot indicates median (black line); the bottom and top edges of the box indicate the 25th and 75th percentiles. Whiskers indicate 1.5 IQR. Outliers are not shown. *Significant different from all (*P* < 0.001). ^#^Significant different from 30 to 45, 45 to 60, 60 to 75, 90 to 105, 200 to 400, ≥400 (*P* < 0.001). ^†^Significant different from 30 to 45, 45 to 60, 60 to 75, 90 to 105, 120 to 150, 200 to 400, ≥400 (*P* < 0.001). ^‡^Significant different from 30 to 45, 45 to 60, 60 to 75, 90 to 105, 105 to 120, 200 to 400, ≥400 (*P* < 0.001).

As shown in Figure 2A, segment length exhibited a large range, from 60 μm for distal vessels to around 2 mm for the main coronaries, with a strongly skewed distribution. Most segments were between 100 μm and 1 mm long. While the segments with smallest diameter were also the shortest, median segment length was remarkably similar in the diameter range between 75 and 200 μm (Figure 2B). Subepicardial terminal segments were longer than midmyocardial and subendocardial ones (Table 1, *p* < 0.001).

**FIGURE 3:**
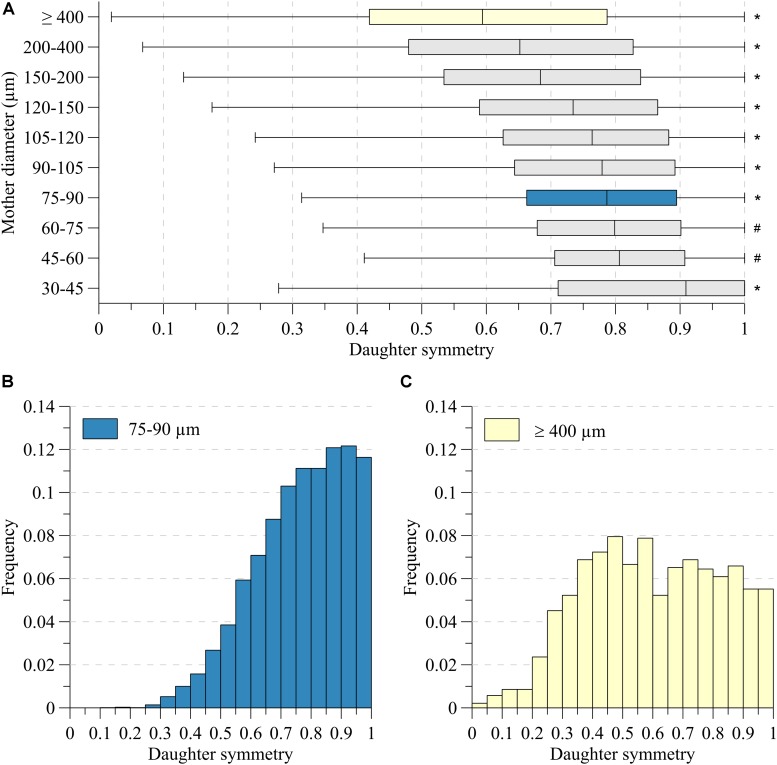
Daughter symmetry for **(A)** ten mother diameter classes. **(B)** Distributions for mother diameter 75–90 μm (blue) and **(C)** ≥400 μm (yellow). *Significant different from all (*P* < 0.05). ^#^Significant different from 30 to 45, 75 to 90, 90 to 105, 105 to 120, 120 to 150, 150 to 200, 200 to 400, ≥400 (*P* < 0.05).

Figure 3A depicts the symmetry of the bifurcations, reflected by the ratio of daughter segment diameters, in the various mother diameter classes. Very asymmetric branches generally reflected consecutive segments of large vessels having small side branches. Here, d_L_ is expected to be close to d_M_ and indeed, the d_L_/d_M_-ratio was significantly larger for such asymmetric nodes (*p* < 0.001). For all diameter classes, in particular for segments larger than 150 μm, node symmetry was highly variable. With decreasing diameter, the nodes became more symmetric, and the dispersion in symmetry decreased slightly, as illustrated by the histograms in Figures 3B,C.

**FIGURE 4:**
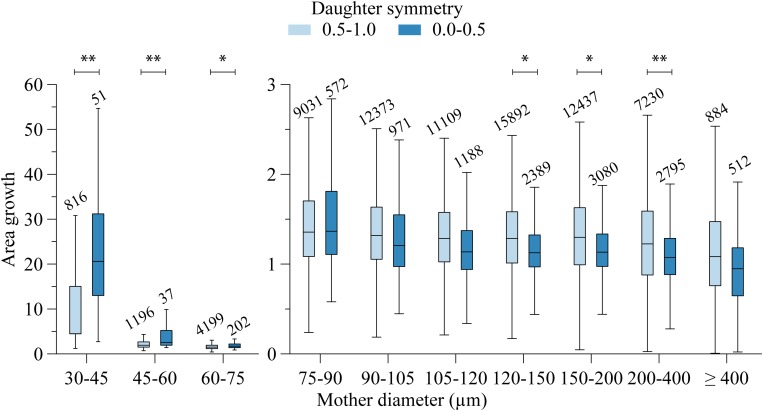
Area growth and mother diameter relation. Per mother diameter class, data are grouped in two daughter symmetry classes. Numbers above the bars indicate group size. Significant difference: ^∗∗^*P* < 0.001, ^∗^*P* < 0.01.

The area growth at bifurcations was also highly variable. For segments larger than 400 μm, on average the cross-sectional area remained stable (median area growth: 1.02). For most other bifurcations, the total cross-sectional area of the daughters was larger than the cross-sectional area of their mother segment. Linear regression showed that area growth (A) increased with decreasing mother diameter (d_M_) and increasing symmetry (S): (*A* = −0.66*d*_*M*_ + 0.34*S* + 1.13,*d*_*M*_ ∈ [75,600]  10^−6^*m*). Even though all coefficients in the fit were highly significant (*p* < 0.001), the low *r*^2^ = 0.047 indicates that mother diameter and daughter asymmetry account only little (4.7%) for the encountered variability. Figure 4 shows this relationship, where the data are grouped in order to comprehensively visualize the effect of both mother diameter as well as symmetry.

### Prediction of Distal Arterial Conductance

**FIGURE 5:**
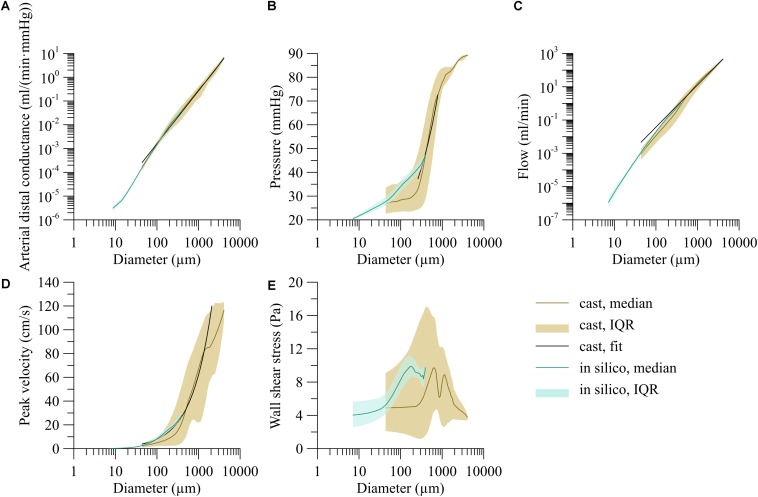
Dependency of hemodynamic parameters on segment diameter for the trees stochastically generated based on the branching characteristics of the human vasculature (*in silico*) and the reconstructed human coronary circulation with extrapolated microcirculation (heart). **(A)** Arterial distal conductance-diameter relation, **(B)** pressure-diameter relation, **(C)** flow-diameter relation, and **(D)** centerline velocity-diameter relation. **(E)** Wall shear stress-diameter relation. Lines indicate the median; areas indicate the interquartile range.

The fifty *in silico* trees, generated from the above described relationships for segment length, daughter symmetry and area growth, on average consisted of 1.22 ± 0.05 million segments. For all trees, there was a linear relation between the log-transformed segment diameter and distal arterial conductance. As the linear regressions were very similar for all trees (coefficient of variation for both coefficients below 0.5%), all *in silico* data were pooled (Figure 5A, turquoise area). For the smallest segments with diameter less than 12 μm, the distal arterial conductance was more variable than for larger segments. Moreover, distal conductance became less dependent on diameter. The increase in distal arterial conductance (in m^3^/(Pa⋅s)) for larger vessels was well predicted by the allometric relation *G*_*is*_ = 1.64⋅10^−3^*d*^2.49^ (d in m; *r*^2^ = 0.98, *p* < 0.001).

### Pressure and Flow Distributions

Hemodynamics in the reconstructed arterial network with stochastically extended downstream conductances were determined in 1,000 simulations of this whole network. Supplementary Figure 1 provides a 3D rendering of the median parameter values derived from the hemodynamic simulations across the segmented vascular segments.

The coefficient of variation of the conductance downstream of a specific end segment over these simulations was 0.11 (median over all end segments). We questioned to what extent this variation in chosen downstream conductances affects the calculated pressure and flow in the complete left coronary network. Table 2 summarizes this analysis. In the stochastic model, pressure in individual segments varied marginally between the simulations, whereas the median coefficient of variation for flow was 7.4%. Hemodynamic simulations with deterministically assigned distal arterial conductances yielded flow and pressure values that fully agreed with the median values of all stochastic simulations. This was the case for two choices for downstream conductance in each measured end segment: based on the power fit above or based on the median value of downstream conductance for similar-sized segments (diameter within 2.5%) in the *in silico* data. This analysis thus reveals that downstream conductance variability affects neither local pressure or its variability, nor median local flow, and adds limited extra variation of local flow in the various diameter classes. For these reasons, further analysis was based on the deterministic model for distal conductance, using the median values in the 2.5% diameter intervals.

**TABLE 2:** Stochastic variation.

	**Variability**				**Agreement with median**			
	**CV**		**MAD**		**Power fit**		**Median G**	
	**Median**	**P95**	**Median**	**P95**	**PCC**	**β**	**PCC**	**β**
Pressure	0.004	0.011	0.10 mmHg	0.36 mmHg	1.000	0.997	1.000	1.000
Flow	0.074	0.104	<0.001 ml/min	<0.01 ml/min	1.000	1.008	1.000	0.999
Wall shear stress	0.074	0.104	0.21 Pa	1.13 Pa	0.999	0.994	1.000	1.000

*Variability of outcome over the 1000 simulations (*Variability*) and agreement with deterministic models (*Agreement with median*). CV, coefficient of variation over the 1000 simulations; MAD, mean absolute deviation over the 1000 simulations; P95, 95-percentile; power fit: comparison to a deterministic model based on a power fit of distal conductance on end segment diameter. Median G, comparison to a deterministic model taking the median distal conductances in 2.5% diameter classes; PCC, Pearson’s correlation coefficient; β, unstandardized coefficient.*

The brown line in Figure 5A represents the median distal arterial conductance for the reconstructed human left coronary tree with extrapolated microcirculation. This line closely matches the *in silico* results by design for small diameters, mostly reflecting terminal segments, yet also agrees well for larger segments [*G* = 1.58⋅10^−4^*d*^−2.22^, d in m, G in m^3^/(Pa⋅s)], *r*^2^ = 0.81; for internal segments the exponent was: 2.18).

Figure 5B shows the pressure as a function of segment diameter. Most of the pressure drop occurred in vessels between 200 μm and 1 mm in diameter whereas only little pressure drop was seen in larger arteries. Despite this trend, the pressure differed greatly between segments of similar diameter. The pressure-diameter relation was *P* = 5.40⋅10^3^*d*^0.60^ (*d* ∈ [250, 800]  10^−6^ m, P in mmHg, *r*^2^ = 0.14).

Blood flow varied with diameter by about a fivefold interquartile range as indicated by the brown area in Figure 5C. Arteries larger than 1 mm carried a median of 36.6 ml/min, about 3,000-times as much blood per vessel as 100 μm segments. An allometric fit of flow (*Q* = *ad*^γ^) over the whole diameter range predicts an exponent γ of 2.53 (*Q* = 5.07⋅10^8^*d*^2.53^, d in m, Q in ml/min, *r*^2^ = 0.83).

As shown in Figure 5D, centerline velocity followed the same trend as blood flow and increased with increasing diameter. The diameter velocity relation was; *v* = 3.00⋅10^4^*d*^0.89^ (d in m, v in cm/s, *r*^2^ = 0.21). Centerline velocity along the epicardial arteries decreased with branching.

The brown area in Figure 5E visualizes the high variability of wall shear stress, particularly for segments between 100 μm and 1 mm. For larger segments, the variation decreased. For segments smaller than 300 μm, median wall shear stress leveled off around 5 Pa.

### Local Myocardial Perfusion

**FIGURE 6:**
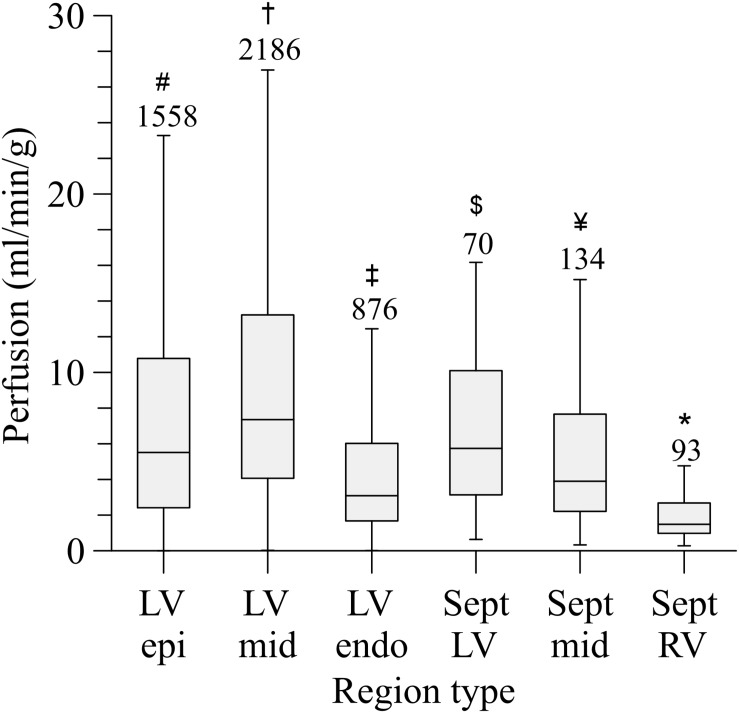
Perfusion distribution per myocardial region. LV, left ventricular free wall; RV, right ventricular free wall; Sept, septum; epi, subepicardium; mid, midmyocardium; endo, subendocardium. Outliers are not shown. Numbers above the bars indicate group size. *Sept RV, significant different from all (*P* < 0.001); ^#^LV epi, significant different from LV mid; LV endo, Sept RV (*P* < 0.001). ^†^LV mid, significant different from LV epi; LV endo, Sept mid; Sept RV (*P* < 0.001). ^‡^LV endo, significant different from LV epi, LV mid, Sept RV (*P* < 0.001), and Sept LV (*P* < 0.01); ^$^Sept LV, significant different from Sept RV (*P* < 0.001) and LV endo (*P* < 0.01). ^¥^Sept mid, significant different from LV mid, Sept RV (*P* < 0.001).

The left coronary circulation perfused in total 358 cm^3^ of myocardium. Truncating the measured tree in the range of 100–200 μm resulted in 4,954 segments perfusing as many territories. The median perfused volume of these truncated segments was 20.2 mm^3^ (interquartile range: 7.2–56.5 mm^3^) with a median perfusion of 5.6 ml/min/g (flow per weight of the perfusion territory), yet with high local heterogeneity (interquartile range: 2.6–10.8 ml/min/g, Figure 6). Despite the higher vascular density, subendocardial perfusion in the left ventricle free wall was lower than midmyocardial and subepicardial perfusion. Septal perfusion from the left coronaries tended to drop from the left toward the right ventricular layer.

## Discussion

In this study, we extensively quantified the branching characteristics of the human coronary circulation, and used these data to predict local hemodynamics, including pressure, flow and wall shear stress, and their variation along the vascular network. These data add to previous work on various animal species and provide a base for a systems analysis of human coronary flow distribution and regulation.

### Previous Data

To the best of our knowledge, no extensive studies have been made on branching patterns and related hemodynamics in the human coronary circulation. Available anatomical studies address specific research questions and provide only limited data that do not allow a translation toward global coronary hemodynamics. This includes the vascularization of the anterior papillary muscle, perfused by the LCA versus RCA (Zajaczkowski et al., 2018), the impact of branching on wave propagation (Rivolo et al., 2016), the effect of side branches on coronary flow (Wiwatanapataphee et al., 2012), and branching patterns of only the large coronary arteries (Hutchins et al., 1976; Seiler et al., 1992; van der Waal et al., 2009; Schoenenberger et al., 2012; Cardenes et al., 2013; Gupta et al., 2013; Medrano-Gracia et al., 2017; Ormiston et al., 2018). In an initial study, we analyzed the presence of collateral connections within and between the perfusion territories in the human heart (van Lier et al., 2016).

While human data are rare, several animal studies do provide quantitative data on coronary artery and microvascular branching patterns. Thus, VanBavel and Spaan analyzed the branching patterns in corrosion casts of porcine hearts, covering the major arteries down to precapillary arterioles (VanBavel and Spaan, 1992). In that work, “branching rules” were derived that relate diameter and length of individual segments, as well as the diameters of mother and daughter segments in nodes. A combination of actual data for the larger vessels and computer-generated distal branches was then used for the assessment of local pressures, flows and their dispersion. A shortcoming in that work was the limitation to topology, ignoring the 3D distribution over the myocardium. More extensive work came from the group of Kassab, who in a series of studies described these characteristics in the arterial, capillary, and venous coronary bed of pigs, also based on corrosion casts, followed by extensive modeling and hemodynamic analyses (Kassab et al., 1993, 1999; Kassab and Fung, 1994; Kalsho and Kassab, 2004; Kassab, 2005; Mittal et al., 2005a, b; Kaimovitz et al., 2008; Namani et al., 2018). Following initial work on manual segmentation of the coronary vasculature based on corrosion casts, our lab has developed an imaging cryomicrotome that allows for extensive 3D recording of branching structures (Spaan et al., 2005) and applied this technique for the study of network characteristics in several species and organs (van Horssen et al., 2010, 2014, 2016; van den Wijngaard et al., 2011; Hakimzadeh et al., 2014; Bedussi et al., 2015; Schwarz et al., 2017), culminating in the current work on the human heart.

### Topology

The branching characteristics were described in terms of segmental diameter-length relations in addition to symmetry and area growth at branch points, as was previously done in porcine hearts (VanBavel and Spaan, 1992). Segmental length was intrinsically variable, reflecting the stochastic nature of the network. There was no clear correlation between segment length and diameter as already has been previously noted for the human right coronary artery (Zamir, 1999). Our previous porcine data predict a mean length of 275 and 619 μm in, respectively, the 90–105 and 200–400 μm diameter class, as compared to the currently observed mean values of 675 and 908 μm. However, in the same animal model Kassab et al. (1993) observed values that are closer to our findings in the current study. The differences between the porcine data may be explained by differences in filling by the casting material. Since very asymmetric nodes occur regularly, the exclusion of very small unfilled side branches would affect a substantial part of the diameter-length relation. Also symmetry was intrinsically variable. Yet, branching was more symmetric and very asymmetric nodes occurred far less in the current human heart as compared to porcine hearts. For example, for large mother diameters (>500 μm) the median observed symmetry ratio was 0.59, in contrast to values below 0.40 for porcine data (VanBavel and Spaan, 1992; Kalsho and Kassab, 2004; Rivolo et al., 2016). Despite the same trend of increasing symmetry with decreasing diameter, this difference was found for all diameter classes, and at least for the larger diameter classes this cannot be attributed to the 30 μm resolution in the current study.

The absence of growth of cross-sectional area in the largest diameter class (mother diameter ≥400 μm) is in accordance with previous observations on human (Zamir, 1999) and porcine data (VanBavel and Spaan, 1992; Kaimovitz et al., 2008), as well as observations from angiographic imaging of the epicardial arteries (Hutchins et al., 1976; Seiler et al., 1992; Schoenenberger et al., 2012; Ormiston et al., 2018). Nodes originating from smaller vessels did show growth of cross-sectional area, implying a decreasing flow velocity toward the microcirculation, as was to be expected. If the diameters of a node would adhere to a scaling law (Eq. 6), area growth (A) at that node would be directly related to the daughter symmetry S by:

(7) $$d_{M}^{\gamma }=d_{L}^{\gamma }+d_{S}^{\gamma }$$

(8) $$A=\frac{1+S^{2}}{{\left(1+S^{\gamma }\right)}^{2/\gamma }}$$

Implying that growth in cross-sectional area increases with increasing daughter symmetry for γ > 2 ($\frac{\partial A}{\partial S}=f\,\left(S,\gamma \right)\,\left(S^{2}-S^{\gamma }\right),f\,\left(S,\gamma \right)>0,S\leq 1$). In our study, symmetric bifurcations indeed tended to show a larger area growth than asymmetric nodes, agreeing with previous observations in the human (Zamir and Chee, 1986) and in the porcine heart (Rivolo et al., 2016). A comparison with prior findings reveals that area growth tends to be higher in the present human heart as compared with similar sized arteries in the porcine hearts (VanBavel and Spaan, 1992), rat and human hearts (Zamir and Chee, 1986), while the variability in area growth was less in the human hearts. Area growth, however, tended to be smaller in the human heart in comparison to values observed in the human cerebral vasculature (Cassot et al., 2010) and in mice hearts (Feng et al., 2018) while following the same general trend of increasing area growth for smaller segments. Estimates of area growth are intrinsically sensitive to precision of diameter measurements, with random errors causing a strong upward bias in the estimate. This holds even more strongly for misclassification of mother versus daughter segments in nodes, and such bias may underlie the high growth in the smallest diameter classes.

### Hemodynamic Predictions

We initially aimed to derive hemodynamic parameters throughout the network by solving the Poiseuille and Kirchhoff equations with assumptions on distal boundary conditions, such as diameter-defined flow or back pressure. Such attempts resulted in highly variable estimates for local pressure and flow that were at variance with the assumed boundary conditions. A better strategy therefore was to assume a diameter-dependent downstream conductance for each outflow segment in the casted vasculature. The generation of this relation by constructing and imputing simulated networks, based on observed and extrapolated branching characteristics and taking variability into account, allowed the hemodynamic analysis of the full, hybrid network.

A comparison of the recorded and imputed part of these networks (brown and blue lines and areas in Figure 5) reveals that the imputed data demonstrated less variability. We conclude from this that we did not fully cover the properties of the recorded data. Aspects that were not included in the simulated parts of the network include arcading segments, trifurcations, and possible deeper correlations in the data, such as correlations between area growth in successive nodes. Such deeper correlations are difficult to discern and quantitate, and we do not expect that including them would strongly affect the predictions of the hemodynamic profiles.

It is now commonly accepted that the major part of the pressure drop occurs over the arterial system, with substantial contribution of arterioles and small arteries. Direct recordings of this pressure distribution are very limited and do not include human hearts. In a classical study in the porcine heart at diastolic arrest and vasodilation, Chilian et al. demonstrated that pressure in 80–120 μm vessels has dropped to 60% and 80% of the perfusion pressure in the subendocardium and subepicardium, respectively (Chilian, 1991). VanBavel and Spaan indeed predicted this range of pressures in their network analysis (VanBavel and Spaan, 1992). However, the current human study predicts a median pressure in 100 μm vessels around 32% of systemic pressure, suggesting that far more of the pressure dissipation occurs in vessels much larger than 100 μm (mainly between 200 μm and 1 mm). This could have been caused by arcades and trifurcations in the data. Also, the presence of daughter segments with a larger diameter compared to their mother could have contributed to this finding. The *in silico* data, with a more standardized vascular pattern, suggest that most of the pressure drop occurs in the range of 30 μm to 300 μm.

The hemodynamic parameters reported in this study for a vasodilated arterial network were slightly higher than measurements in human subjects during hyperemia. Flow velocities in the range of 10–30 cm/s are reported in angiographically normal epicardial coronary arteries at rest and 60–100 cm/s at hyperemia and are highly variable between individuals (Kern et al., 1990; Ofili et al., 1995). Flow velocities tend to be lower in the left anterior descending and left circumflex coronary artery compared with the left main coronary artery (Kern et al., 1990). The high flow velocities found in our study were associated with high levels of perfusion (median: 5.6 ml/min/g). Perfusion as measured with positron emission tomography typically increases from 0.6–1.2 ml/min/g at rest to values between 1.9 and 5.0 ml/min/g during hyperemia, with higher values found in females (Sdringola et al., 2011; Murthy et al., 2018). In animal models, regional differences in perfusion were reported with generally equal or higher perfusion at the subendocardium compared to the subepicardium (Feigl, 1983). For the human heart in the present study we found the opposite. Since the simulations were based on data obtained in a diastolic heart, the results resemble hemodynamics in a diastolically arrested heart. Furthermore, the influence of cardiac contraction and myocardial tone were not taken into account in our hemodynamic model. In the beating heart, systolic flow in notably the subendocardium is impaired by the contracting surrounding myocardium (Hoffman and Spaan, 1990). In a model study, Namani et al. showed that not only is flow higher under passive conditions than under autoregulation, but that ignoring the interaction between vessels and the surrounding myocardium indeed results in increased flow and thus perfusion estimates (Namani et al., 2018). Bache et al. found an increase in the subendocardium/subepicardium perfusion ratio with decreasing heart rate at maximal vasodilation (Bache and Cobb, 1977). This together with topology artifacts, ignoring extra resistance in bifurcations or possibly a comparatively low prescribed capillary pressure could have influenced our hemodynamic calculations.

### Allometric Description of Topology and Hemodynamics

Allometric (power law) relations have regularly been used to describe relations between physiological and anatomical parameters (Thompson, 1942), including the coronary circulation (Kassab, 2005; Molloi and Wong, 2007; Le et al., 2008; Huo and Kassab, 2009b, a, 2016). The arguably best known relation is “Murray’s law” (Murray, 1926) that, based on cost minimization principles, relates flow in a blood vessel to the cube of its diameter. In Poiseuille flow, this relates to wall shear stress being constant along the vasculature. Murray’s law certainly is not universally valid, and alternative allometric relations have been derived from various optimality principles, including space-filling requirements (Cohn, 1954; West et al., 1997), relating properties of stems to corresponding crowns (Huo and Kassab, 2009a), optimization of total conductance (Razavi et al., 2014), and application of constructional laws (Miguel, 2016). The postulated scaling exponent typically ranges between 2 and 3.

Our observation of constant cross-sectional area in the larger coronaries is in accordance with several other studies (VanBavel and Spaan, 1992; Mittal et al., 2005b), suggesting an exponent close to 2 rather than 3 for large coronaries. The scaling exponent γ can be derived from area growth by fitting Equation 7 or alternatively by fitting any reformulation of Equation 6. There are various possible optimization strategies, resulting in different estimates for γ, which are furthermore strongly influenced by extreme values. It was therefore difficult to thoroughly quantitate γ based on branching. In general, the scaling exponent increased with decreasing diameters, typically from a value close to 2 for the large segments toward values larger than 3 for small segments. Rivolo et al. also observed the same trend in the porcine heart, with a γ increasing from approximately 2.25 for large vessels to values around 4 for vessels smaller than 100 μm (Rivolo et al., 2016). For the human heart in the present study, the scaling exponent in the diameter-flow relation decreased from 3.21 in the smallest vessels to 2.55 in the major vessels, and shear stress depended biphasically on diameter, having its peak at 630 μm. Van der Giessen et al. found an exponent of 2.55 based on biplanar angiography of human epicardial arteries for the flow-diameter relation (van der Giessen et al., 2011), which matches our results. The exponents in the human heart appear to be higher than the ones observed in porcine hearts. VanBavel and Spaan also described non-constant exponents, increasing from 2.35 for segments larger than 200 μm to 2.82 for diameters smaller than 40 μm (VanBavel and Spaan, 1992). Mittal et al. used morphometric data of the entire porcine coronary arterial tree down to the first capillary branch to conduct hemodynamic analysis and determined a flow–diameter power law relation with exponent 2.2 (Mittal et al., 2005a). Despite these deviations from Murray’s and other invariant scaling laws, downstream conductance scaled with diameter to the power 2.22 over a large range. Common to all invariant allometric relations is that they postulate a general optimality principle. However, as derived by Uylings, different exponents are ideal for different flow types (ranging between 2.33 for turbulent to 3.0 for laminar flow) (Uylings, 1977). Similar effects have been shown for rheological variation (Miguel, 2016) and flow pulsatility (Rivolo et al., 2016). Flow in the coronaries is pulsatile and near-Newtonian whereas laminar, non-pulsatile flow subject to shear-rate dependent viscosity is characteristic for the small vasculature. These differences together with other influences mainly affecting the larger vessels such as areas of low or oscillating wall shear stress (Schoenenberger et al., 2012) and wave reflections (Reneman et al., 2006; Rivolo et al., 2016) may lead to shifting optimal branch relations as we have observed. Altogether, while it is tempting to use allometric descriptors, reality seems a bit more complex and the deviations may lead to substantial differences in physiological parameters such as shear stress and local pressure.

### Limitations

A series of image processing steps was required to translate the image stack to a network representation. These included segmentation of the arterial bed, 3D skeletonization, and diameter estimation. This was a major challenge, due to the large difference in vascular diameter between the major branches and the 30 μm smallest vessels that were included. Manual and automated correction was needed. The choices here were based on comparison of the original images to the vascular network representations as well as on common sense. Thus, a few interruptions in cast filling of the major vessels needed to be corrected manually. Very short triangular loops were clearly the result of errors in skeletonization and were pruned to simple paths. Likewise, spurious side branches were removed and some regularization of diameter along vascular paths was included. Since as many as 202,184 segments were included in the final representation, some level of error in the topology and diameters remains unavoidable. However, while it remains necessary to further test and improve the procedures, we do believe that the current work provides an adequate translation from the image stack to the graph representation.

The heart was imaged at 30 μm resolution. Moreover, care was taken not to fill the vessels toward the capillary bed, as this would have resulted in strong background fluorescence. Meanwhile, data on the more distal arterioles were needed for the hemodynamics analysis. We therefore extrapolated branching characteristics when simulating the network toward 5.0–7.5 μm segments. Future work should provide branching characteristics of also the smallest arterioles. While it will not be feasible to fully cover such branching in the whole heart, microscopic data from much smaller tissue samples could be imputed into the simulated networks, improving reliability of the hemodynamic analysis.

The current study is based on a single heart from an elderly patient having atherosclerosis and may not be representative for a normal heart from a person in the same age category or for a healthy young heart. Even though Chen et al. showed in a mice model that the scaling exponent is not affected by aging (Chen et al., 2015), we acknowledge that this may limit the current work. Yet, human hearts rarely become available for this purpose and the whole procedure from filling to graph representation is extremely labor- and computer-intensive. While it will not be feasible to perform studies such as the present one on large numbers of human hearts, inclusion of more hearts is needed to draw conclusions on reproducibility and dependence on age, sex and morbidities. The current study provides a pipeline for doing this work, and with further optimization, automation of the procedures, and improved computer power the inclusion of more samples should become feasible in the near future.

Further limitations of the work include the assumption of Poiseuille flow, the exclusion of the capillary and venous bed in the hemodynamics calculations, and the exclusion of the wave transformation effect. We presume that these assumptions have not substantially altered our main findings. We also were not able to analyze the right coronary artery perfusion territory due to insufficient quality of the vascular filling and subsequent segmentation.

### Future Work and Application of the Current Data

An important next step will be the direct quantification of also the most distal vessels, as well as improved routines for image processing and imputation strategies based on a deeper analysis of the data, in addition to extension of the data set.

It has become clear over the years that CAD is not limited to the epicardial vessels. Rather, intra-myocardial arteries and arterioles are also affected, as evidenced by impaired endothelial responsiveness and alterations in vascular structure, caliber and resistance (Crea et al., 2014). Moreover, the vasculature adapts, for better or worse, to the presence of a proximal stenosis and the associated reduced pressure, vascular tone, and hyperemic flow (Siebes et al., 2004; Verhoeff et al., 2005; Sorop et al., 2008). Local perfusion and perfusion reserve are known to be heterogeneous in animal models and healthy humans (Bassingthwaighte et al., 1989; Austin et al., 1990; Chareonthaitawee et al., 2001), with heterogeneity increasing at smaller length scales. Depending on the adaptation of the coronary bed, developing CAD and its effects on the microcirculation may well affect such dispersion of local perfusion and reserve, leading to local ischemic zones. A framework such as presented here could help understanding the relation between microvascular structure and function and the local perfusion and perfusion reserve. We therefore foresee application of the current data and analyses in systems approaches of the coronary circulation, leading to new experimentally testable hypotheses on microvascular adaptation in CAD. In addition, the current study may add to the modeling required *in silico* clinical trials on new drugs and devices, in extension to the current *in silico* work on drug-eluting stents (Karanasiou et al., 2018) and in analogy to work currently done in acute ischemic stroke (Konduri et al., 2018).

### Conclusion

We have presented a processing pipeline and extensive data on branching characteristics and predicted hemodynamics of the human coronary circulation. Our findings provide a base for further modeling, including incorporation of vasomotor responsiveness, structural adaptation and their effects on the balance between oxygen demand and supply in health and CAD.

## Data Availability Statement

The datasets generated for this study are available on request to the corresponding author.

## Author Contributions

JS, ML, and EV designed the study. JS, JW, and EV developed the methods and software. JS and ML carried out the study and statistical analysis. All authors critically reviewed the manuscript and approved the final version.

## Conflict of Interest

The authors declare that the research was conducted in the absence of any commercial or financial relationships that could be construed as a potential conflict of interest.
