# Supplementary figures and images for: Topologic and Hemodynamic Characteristics of the Human Coronary Arterial Circulation
Source: Front Physiol. 2020 Jan 23;10:1611. doi: 10.3389/fphys.2019.01611 (PMC6989553; doi:10.3389/fphys.2019.01611)

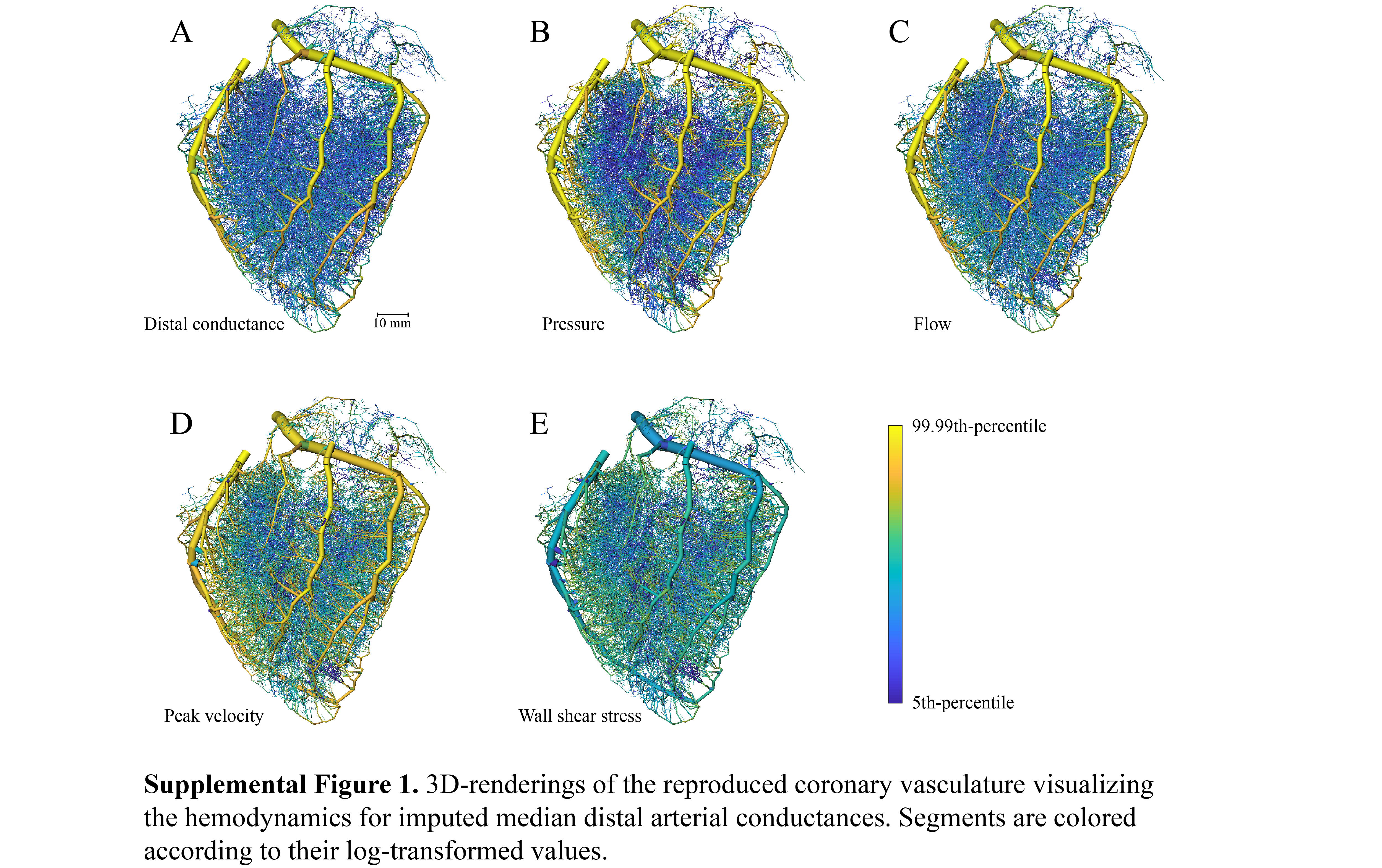

Supplement: Supplementary file 1 [file Image_1.PNG]
